# Persisting Transglutaminase 6 Antibodies in Neurological Gluten‐Related Disorders

**DOI:** 10.1002/ana.78020

**Published:** 2025-08-25

**Authors:** Iain D. Croall, Marios Hadjivassiliou, David S. Sanders, Kevin Teh, Alberto M. Biancardi, Nick Trott, Nigel Hoggard

**Affiliations:** ^1^ School of Medicine and Population Health University of Sheffield Sheffield UK; ^2^ Department of Neurology Sheffield Teaching Hospitals NHS Foundation Trust Sheffield UK; ^3^ Academic Unit of Gastroenterology Sheffield Teaching Hospitals NHS Foundation Trust Sheffield UK

## Abstract

**Objective:**

Gluten‐related autoimmunity can cause neurological disease, although the best way to diagnose and monitor such patients is unclear. Serological testing for antibodies against transglutaminase 6 (TG6) has been proposed; however, this is not widely available in clinical practice. Using longitudinal data from patients attending a specialist neurological center with routine TG6 testing, this observational study explores how antibody history relates to brain atrophy, cognition, and quality of life.

**Methods:**

Serological records of patients with gluten‐related neurological disease were collected alongside clinical brain magnetic resonance imaging. Patients were recruited to undertake questionnaires that assessed/included chronic symptom severity, the hospital anxiety and depression scale, the SF‐12, and the Biagi scale for gluten‐free diet adherence. Volunteers were offered the cerebellar cognitive affective syndrome scale for cognitive testing. Primary analyses focused on patients with ≥5 years of serology (n = 462), and related TG6 history to available clinical outcomes (primary analysis range 89–104).

**Results:**

Patients with a previous positive immunoglobulin A (IgA) TG6 result reported greater depression, symptom severity, and poorer physical functioning. IgA TG6 antibody exposure was correlated with regional brain atrophy (age‐corrected). Greater self‐reported gluten‐free diet adherence significantly predicted a recent negative IgA TG6 test. Subgroup analyses replicated multiple findings in patients with and without celiac disease.

**Interpretation:**

TG6 testing can identify patients at risk of accelerated brain atrophy, poorer physical functioning, and worsened mental health. IgA TG6 should be used as a diagnostic and monitoring test for patients with relevant neurological presentations, while achieving negative serology with a strict gluten‐free diet should be the goal. ANN NEUROL 2026;99:274–282

Gluten‐related disorders are autoimmune. The pathogenesis of celiac disease (CD) involves the production of autoantibodies to transglutaminase 2, more commonly called tissue‐transglutaminase (TTG).^1^ Anti‐transglutaminase 3 antibodies have been linked to dermatitis herpetiformis, a gluten‐mediated autoimmune skin condition where the target has been shown to be epidermal transglutaminase 3.^2^ More recently, anti‐transglutaminase 6 antibodies (TG6) have been discovered and associated with gluten‐related neurological disease.^3^ This describes a range of manifestations^4^ that are defined by a response to a strictly maintained gluten‐free diet. The most common and best studied manifestation is gluten ataxia,^5^ associated with Purkinje cell loss.^6^ The second most common manifestation is gluten neuropathy, an axonal length‐dependent neuropathy.^7^ The third common manifestation is gluten encephalopathy, a combination of headache and accelerated cerebral white matter disease.^8^

Since their discovery, investigations have shown an overrepresentation of TG6 antibodies in patients with sporadic ataxia,^9^, ^10^ supporting that many of these cases may be driven by gluten autoimmunity. Some studies have failed to demonstrate heightened TG6 in similar cohorts, but often suffer from low power in relevant analyses.^11^ Few reports have sought associations between TG6 and clinical outcomes. In newly‐diagnosed patients with CD presenting to the gastroenterology department, 40% are TG6‐positive, and this subgroup shows atrophy of the cerebellum and thalamus.^12^ TG6 is widely expressed throughout the central nervous system, but particularly in such brain and regions that contribute to motor function.^13^, ^14^ Mutations in the TG6 protein encoding gene have been linked to a form of spinocerebellar ataxia.^15^ TG6 antibodies may therefore be both a powerful diagnostic tool and component of the neurodegenerative process in these patients.

The historical lack of a specific serological biomarker for gluten‐related neurological disease may have led to misconceptions around the condition. The emergence of TG6 serology offers an explanation for why gluten‐related neurological dysfunction can be found in some patients with classical CD, but not in others. These conditions are likely to cluster, as with dermatitis herpetiformis, being driven by gluten, but underpinned by distinct autoimmune mechanisms directed at different types of transglutaminases (TG2, TG3, and TG6). Supporting this, literature over the past 2 decades has shown an overrepresentation of related gluten antibodies in sporadic adult‐onset ataxia,^16^, ^17^ which symptomatically improves when a gluten‐free diet (GFD) is strictly adhered to.^18^, ^19^

In the UK, National Institute of Care Excellence guidelines now advise testing for CD in patients with otherwise unexplained ataxia/neuropathy.^20^ However, such recommendations do not acknowledge gluten‐mediated ataxia/neuropathy without a co‐diagnosis of CD. TG6 positivity can occur in neurological patients with or without CD.

In this cross‐sectional study, we investigated longitudinal serological and neuroimaging data, alongside cognitive and quality‐of‐life outcomes from patients attending a specialist center for gluten‐driven neurological disease. History of TG6 is examined with respect to disease severity/progression, hypothesising its presence is associated with various forms of neuropsychological deficit in patients with or without CD. This would further validate gluten as being a driver of disease in these patients, and highlight the TG6 test as a clinical tool that merits wider adoption into clinical practice.

## Methods

### Clinical Setting

The Sheffield Ataxia Center (UK) includes a specialist clinic that has treated forms of neurological gluten sensitivity since 1996. Patients may or may not have CD. Those with CD are often referred from gastroenterology if neurological symptoms become a concern. Those without CD present with sporadic neurological symptoms and are diagnosed with a suspected gluten‐mediated mechanism via exclusion of other causes and seropositivity for gluten‐related antibodies. Some of this latter group may be found to have undiagnosed CD via these blood test investigations, but many do not, and it is the presence of other gluten antibodies that confirm their gluten‐related diagnosis (either to native gliadin or TG6). At this stage, the GFD is advised (with support) for all patients, and follow‐up clinical appointments are made at routine intervals (commonly at 3, 6, or 12 months). Further care involves the continuous monitoring of all gluten‐related antibodies as a means of measuring dietary success and ongoing immune reactivity. Brain magnetic resonance imaging (MRI) scans are also performed wherever considered clinically necessary. Clinic codes in hospital records identify all patients who have attended the clinic.

The ethics for the study was approved by the Health Research Authority via the London Bridge Research Ethics Committee (REC reference 21/PR/1356). Patients who completed questionnaires or cognitive testing provided informed consent. Retrospective clinical data was otherwise anonymized and acquired for study as per the ethical agreement.

### Data Collection

Existing serological test data were collected from hospital trust databases. All patients in the 20 years before the initial data request (between 2002 and 2022) who had attended these clinics and at some point returned a positive gliadin antibody result were included, matching the historical, primary diagnostic method for neurological gluten‐related disorders regardless of CD status. Linked longitudinal serology and brain imaging data from these patients were used for study analyses. These data were anonymized. Enzyme‐linked immunosorbent assayTG6 testing (Zedira, Darmstadt, Germany) was implemented into routine clinical care from 2019. Both immunoglobulin (Ig)A and IgG tests were performed, with results reported as raw titers and positivity defined as >4.1/5.2 for IgA/IgG, respectively.

The study also gathered current outcome data from patients via cognitive performance testing, and a holistic set of self‐reported variables pertaining to dietary adherence, symptomatology, mood, and quality of life.

For the self‐reported information, postal questionnaires were used. Patients with an upcoming clinical appointment in the following weeks were sent a package in the post, which included a questionnaire with a prepaid return envelope. This questionnaire mixed bespoke items with validated inventories. The full questionnaire is available in Supporting Information, but details pertinent to the current study included:

- Overall daily symptom severity (via visual analog scale);
- Self‐reported GFD dietary adherence assessed via the Biagi score^21^;
- Assessment of depressive and anxious traits via the Hospital Anxiety and Depression Scale (HADS)^22^;
- Assessment of quality of life, via the “physical” component of the SF‐12 (the “mental” component was also collected, but not used in favour of the HADS outcomes).^23^

For the cognitive assessment the Cerebellar Cognitive Affective Syndrome‐Scale (CCAS‐S)^24^ was used. This was selected given that the primary complaint for patients was anticipated to be ataxia or to involve other cerebellar features. A trained study team member (I.D.C.) administered these.

Postal questionnaires and cognitive testing sessions were in this way offered to patients on a rolling weekly basis until September 2023, when all data collection for the present study analyses ceased.

### Further Data Preparation

*Serology*: Longitudinal serological information relating to TG6 were summarized. For each patient the following were determined:

- If they had ever tested positive (assuming they had at some point received at least 1 TG6 test).
- An experimental variable termed their antibody “exposure.” This is intended to be analogous to smoking pack years. For each patient TG6 test, the duration (in days) since the last test of the same immunoglobulin type was multiplied by the raw titer value. The sum of these per‐patient was the antibody “exposure.” In this way, patients with persistently high titers would be distinguished from those with moderately high levels, whereas those with “negative” titers would also contribute a proportionately low, but non‐zero statistic to analyses. The first test instance for each patient was given a “dummy” duration value of 1 day.

These variables were calculated separately for TG6 IgA and IgG immunoglobulin forms. If a patient had CD was determined by if they had ever returned a positive TG2 or endomysial antibody test.

Individuals with at least 5 years of serological data were selected at this stage for primary analyses. This ensured that patients were only included where there was an ongoing clinical need to monitor gluten antibody status. Although the maximum duration available for TG6 would always be limited by its testing start date in 2019, this also meant that accuracy of the TG2 and endomysial testing would benefit from an improved longitudinal profile.

*Brain imaging*: The objective of the brain imaging analyses was to assess regional rates of brain atrophy. Volumetric T1‐weighted MRI scans, which were performed using the same sequence and scanner, were carried out across all patients (Ingenia 3T; Philips, Eindhoven, the Netherlands; 3D T1, 0.937mm^2^ × 1 mm resolution). Patients with 2 such acquisitions, which were at least 5 years apart, were identified (those with the greatest temporal distance being selected). These images were inspected for quality, and any with problematic artefacts were discarded.

T1 scans were processed using conventional analysis tools provided by the FSL package.^25^ The SIENA pipeline^26^ was used to produce “flow” images that underwent a voxelwise analysis investigating brain atrophy patterns between scan A and scan B. Supplementary subcortical structure volumetric analysis was performed using the FIRST pipeline.^27^ Atrophy between these regions was then calculated by taking the volume difference between each scan. In all instances, overall atrophy variables were further divided by the time between each scan (expressed in years), per patient, so that the data being analyzed statistically was effectively the percentage yearly loss of volume.

### Statistical Analyses

Statistical tests were performed in SPSS version 28 (IBM, Armonk, NT, USA), except for the voxelwise SIENA test, which is run by permutation analysis using FSL's “Randomize” tool^28^ (with 10,000 permutations and threshold‐free cluster enhancement applied).

All variables were visually inspected for normality to determine appropriate statistical models. IgA and IgG TG6 data (if ever positive and antibody exposure) were separately analyzed in groupwise testing/correlations against chronic symptom severity, HADS depression score, HADS anxiety score, SF‐12 physical functioning score, CCAS‐S total passes, and via the voxelwise SIENA analysis against the rate of brain atrophy. Post‐hoc analyses were performed in relevant subvariables as appropriate to establish underlying patterns of effect (eg, brain regions of interest following any first indications of regional effects ascertained from the voxelwise SIENA analysis). Supplementary tests sought replication of significant TG6 versus outcome comparisons in patient subgroups defined by the presence of CD.

The Biagi score was used to determine dietary “adherent” (≥3) and “non‐adherent” (≤2) individuals. Primary outcomes that held a significant relationship with TG6 were compared between Biagi groups to explore if self‐reported dietary adherence predicted these. Finally, χ^2^ analysis compared the outcome of the most recent IgA/IgG TG6 test result between Biagi groups. Only patients who had at some point tested positive for the given antibody were included, so that a recent negative result could be indicative of a treatment effect rather than a persistent baseline. This analysis was sought across the whole patient sample (including those with <5 year history).

### Role of the Funding Source

Study funders had no role in the study design, data collection, analysis, interpretation, writing of this report, or decision to submit for publication.

## Results

### Overview

**FIGURE 1:**
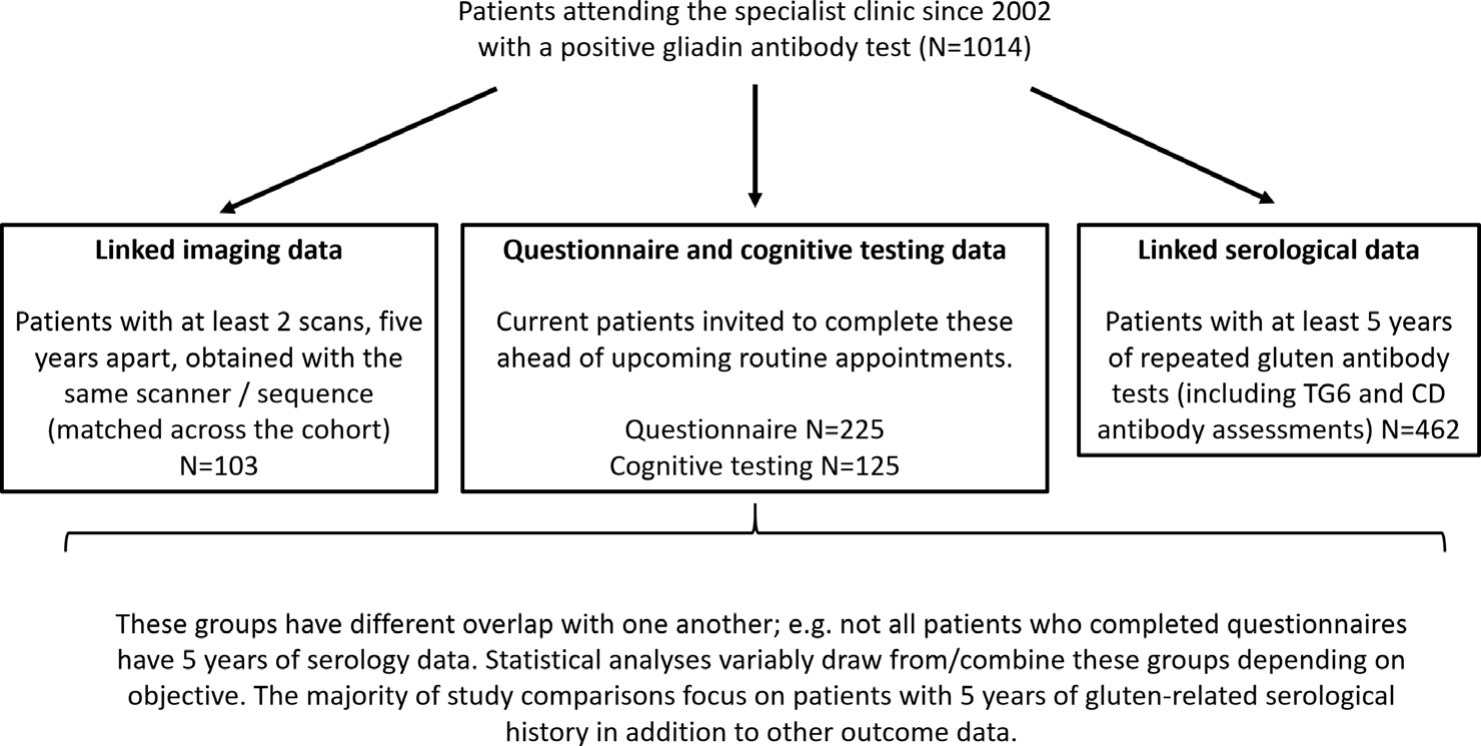
Flowchart summarizing patient selection and sample sizes across different study groups.

Figure 1 presents a flowchart that summarizes participant selection and groupings. The final sample sizes for the different data streams of the study were as follows. Serology data over 5 years: n = 462, compatible imaging data over 5 years: n = 103, completed CCAS‐S: n= 125, completed dietary, mood, and quality of life questionnaires: n = 225. At the time of completing the questionnaire, patients were on average aged 61.2 ± 14.1 years, and 64.2% were women.

### Serology

All following analyses concern only patients with at least 5 years of serological data unless otherwise stated. The rate of TG6 positivity was 62.9%/34.3% for IgA/IgG, respectively. All patients were advised to undertake a strict GFD. The χ^2^ analysis showed a significant relationship between seropositivity for both forms (*p* < 0.001); 75.3% of IgG‐positive patients were at some point IgA‐positive, whereas 58.9% of IgA‐positive patients were at some point IgG‐positive. Age (at time of returning a questionnaire) was not different between patients with and without historical TG6.

### TG6 Versus Questionnaire and Cognitive Data

**FIGURE 2:**
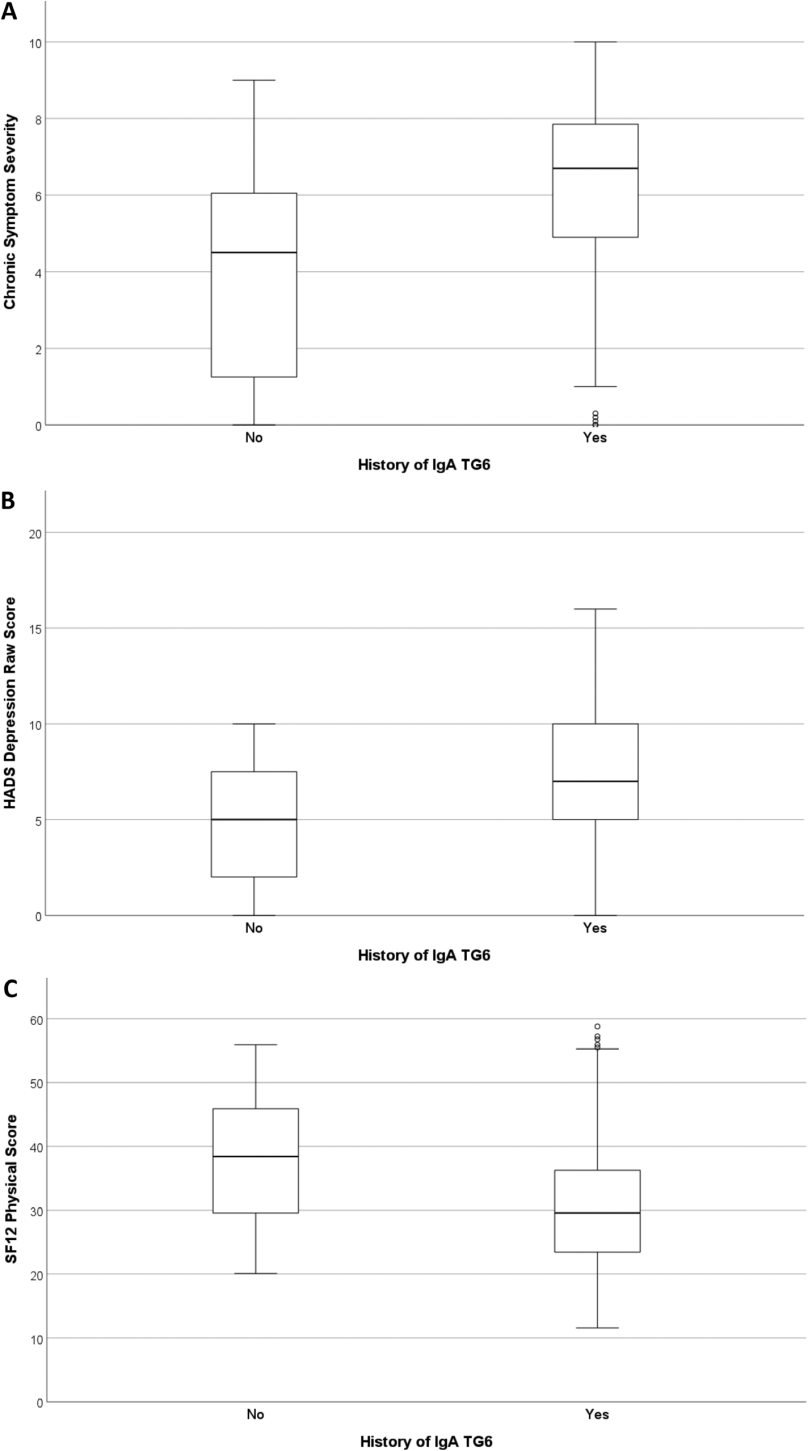
Boxplots visualising significant results where history of a positive immunoglobulin A (IgA) transglutaminase 6 (TG6) result predicted poorer self‐reported patient outcomes. HADS = Hospital Anxiety and Depression Scale.

Table 1 summarizes groupwise testing between patients based on IgA TG6 history with respect to quality of life measures. Age was not correlated with any outcome. Having ever returned a positive IgA TG6 significantly predicted poorer symptom severity (*p* < 0.001), depression (*p* = 0.01), and physical functioning scores (*p* = 0.013). These findings are shown in Figure 2. IgA TG6 exposure additionally showed a positive Spearman's correlation with symptom severity (*r* = 0.232, *p* = 0.023). IgG TG6 positivity and exposure returned no associations.

**TABLE 1:** Summary of Groupwise Analyses Comparing Patients Based on the Existence of a Positive Immunoglobulin A TG6 on Self‐Reported Outcomes

Outcome (sample size in analysis)	IgA TG6‐positive group mean ± SD	IgA TG6‐negative group mean ± SD	*p* value
Chronic symptom severity (n = 95)	6.2 ± 2.7	4.1 ± 2.7	**<0**.**001**
HADS anxiety raw score (n = 102)	8.1 ± 4.9	7.0 ± 4.5	0.312
HADS depression raw score (n= 104)	7.1 ± 3.8	4.8 ± 3.2	**0**.**010**
SF‐12 physical functioning (n = 89)	31.5 ± 12.4	38.8 ± 10.9	**0**.**013**

Symptom and physical scores were non‐parametric; groupwise tests performed by Mann–Whitney *U* test. Significant *p* values are in bold.

HADS = Hospital Anxiety and Depression Scale; TG6 = transglutaminase 6.

Across the whole cohort, the rate of different outcomes from the Cerebellar Cognitive Affective Syndrome‐Scale (CCAS‐S) were as follows. Pass: 16%; Possible CCAS: 18.4%; Probable CCAS: 26.4%; Definite CCAS: 39.2%. The number of CCAS‐S passes was not significantly different between patients who had ever tested positive for either immunoglobulin A or immunoglobulin G transglutaminase 6, and did not correlate with exposure scores.

### TG6 and Brain Imaging

**FIGURE 3:**
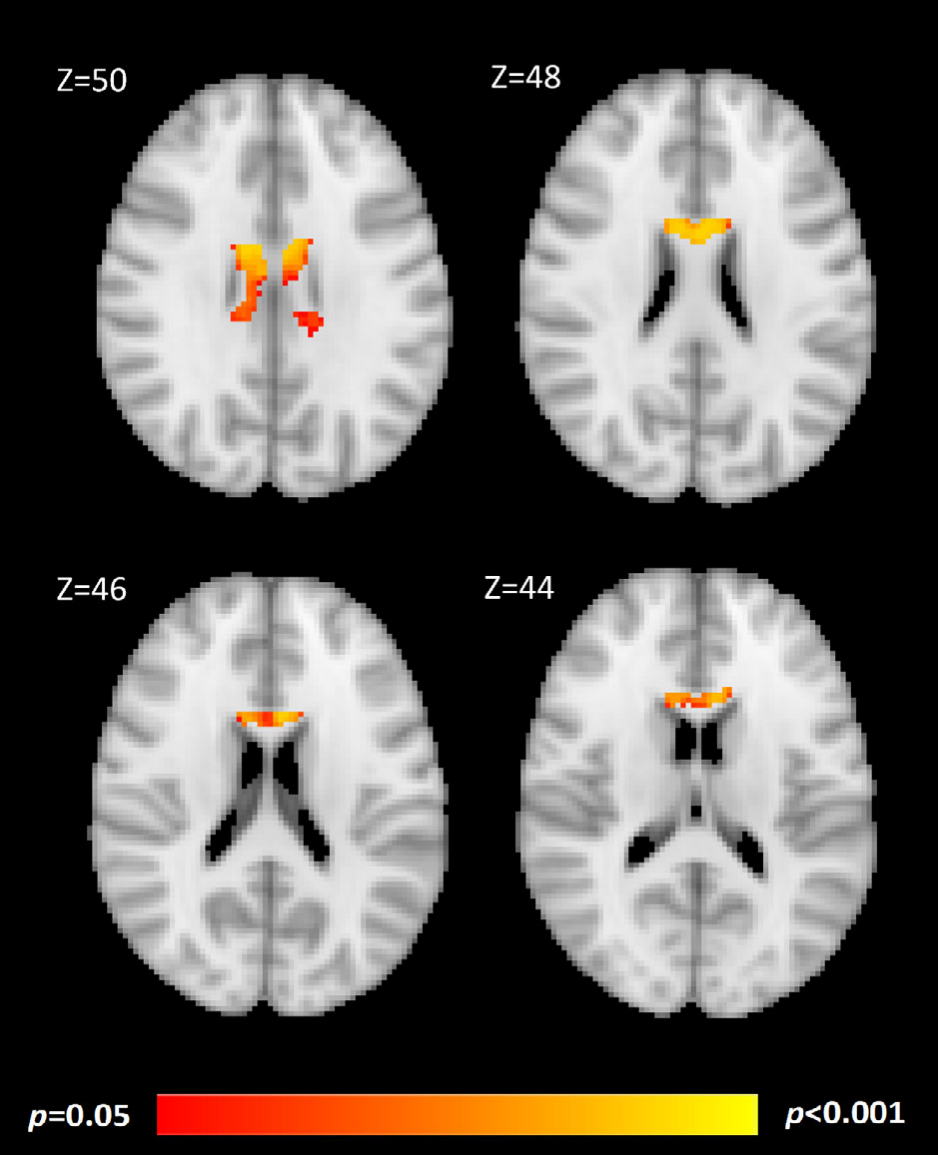
Brain regions where immunoglobulin A transglutaminase 6exposure (ranked) holds a negative correlation with rate of atrophy in patients without celiac disease, corrected for age. Areas highlighted in red/yellow clusters have a faster rate of atrophy with greater immunoglobulin A transglutaminase 6 exposure in this group. The template brain used for visualization is the MNI152 (2 mm) with Z slice co‐ordinates given for reference. [Color figure can be viewed at www.annalsofneurology.org]

Brain imaging analyses are age‐corrected, as age (at the time of the second scan) had a significant correlation with the rate of global atrophy, as determined from the primary SIENA data (*r* = −0.412, *p* < 0.001). Voxelwise analysis showed IgA TG6 exposure to be associated with a faster rate of atrophy in brain regions, which prominently included the genu of the corpus callosum and superior regions of the lateral ventricles (Fig 3).

### CD Versus Non‐CD Subgroup Analyses

See Supporting Information for analyses, which sought replication of the above significant findings in patient subgroups based on the presence of CD. Briefly, results were broadly replicated in both subgroups or approached significance (*p* < 0.1) in a manner consistent with reduced power given the smaller sample sizes. The exception was brain atrophy results, which were replicated only in patients without CD. Further post‐hoc analysis in this subgroup showed subcortical gray matter/brainstem atrophy to be driving voxelwise IgA TG6 findings.

### Gluten‐Free Dietary Adherence and TG6/Outcomes

Comparing groups defined by Biagi adherence between previously significant outcomes found this did not significantly predict whole brain atrophy, SF‐12 physical functioning, or chronic symptom severity, but adherent patients did return significantly lower depression scores (6.15 ± 3.66, n = 82) than non‐adherent patients (8.42 ± 4.10, n = 19, *p* = 0.019). In patients who had ever been IgA TG6‐positive, the “adherent” group was significantly more often negative for this antibody on their most recent test (43.2%) than those who were “non‐adherent” (21.4%, *p* = 0.034). The total sample size for this comparison was n = 139. IgG TG6 positivity did not show a statistically significant change with respect to Biagi scores.

## Discussion

In this large, longitudinal study, we investigated the role of TG6 antibodies in patients with gluten‐related neurological disease by analyzing clinical brain imaging and other outcomes describing patient wellbeing. Our results show that the presence of IgA TG6, historically or ongoing (despite GFD), is linked to a number of adverse consequences: rate of brain atrophy, as well as self‐reported symptom severity, mood, and physical functioning. Furthermore, in patients who had previously tested positive, greater gluten‐free dietary adherence was shown to predict a recent negative IgA TG6 result. This study validates the utility of TG6 testing in patients with gluten‐responsive neurological problems, regardless of CD status.

Gluten is known to drive neurological disease in some people genetically at risk, but how exactly this is best diagnosed and if a co‐diagnosis of CD is necessary has remained contentious. In 2008, a novel gluten‐related antibody was discovered against TG6,^3^ a member of the transglutaminase enzyme family, found predominantly in the central nervous system.^13^ Various studies have since linked TG6 positivity to gluten‐related neurological disease and clinical outcomes. It predicts lower thalamic volume in patients with newly‐diagnosed CD,^12^ and is prevalent in patients with gluten ataxia^9^, ^10^ and neuropathy.^29^ Some studies have shown that achieving negativity of all gluten‐related antibodies improves MRI brain outcomes, such as recovery of cerebellar N‐acetylaspartate,^19^ slowing of regional brain atrophy,^30^ or improved clinical observations.^18^ Previous research, however, suffers variably from heterogenous antibody “positive” groups (combining multiple gluten‐related antibodies), small sample sizes, and unblinded outcome measurements.

To properly validate the utility of TG6 testing for suspected gluten‐related neurological disease, the current study utilized historical clinical data from a specialist center, which routinely monitors TG6. From this large cohort, retrospective contemporaneously acquired blood test results and brain MRI were analyzed. For additional characterization of patient outcomes, cognitive testing was performed alongside questionnaires assessing dietary compliance and quality of life measurements. This investigation, therefore, has considerably greater power, scope, and clinical validity than any before it.

We showed that a history of IgA TG6 is related to a number of adverse outcomes in these patients; poorer symptom severity, mood, and physical functioning. By analyzing isoforms separately, the results from this study should help guide further research and treatment to be more targeted. Our results agree with previous research in sporadic ataxia, which found IgA, but not IgG TG6, to be raised in a cohort of patients with sporadic ataxia.^9^ This might reflect the relatively shorter half‐life of IgA versus IgG in the circulation. IgA may act as a more dynamic and responsive biomarker of ongoing gluten exposure(s) in the gut, also explaining why recent IgG test results did not appear linked to self‐reported dietary adherence.

IgA TG6 exposure was linked to a faster rate of brain atrophy. Voxelwise and post‐hoc/supplementary investigations showed the affected regions to be the brainstem, accumbens, and area surrounding the lateral ventricles, particularly in patients without CD. Of note, atrophy of the cerebellum was not shown in the analysis pathway to be linked to IgA TG6. These findings, therefore, implicate the antibody to be relevant to more cerebral pathology, which has been shown in gluten‐related disease in a number of previous reports.^8^, ^31^, ^32^ It should also be highlighted, however, that automated image segmentation software performs worse with increasing abnormality of a target area.^33^ As cerebellar atrophy is severe in many of these patients, it is likely that data noise relating to that area is high, and this may have led to false negative results. Cerebellar atrophy has been shown or suggested to be linked to TG6 in previous studies of patients with CD.^12^, ^30^ Otherwise, the benefit of automated software used in these analyses is reproducible and eliminates any operator bias. Brain imaging analyses also reached significance using the “exposure” variable, supporting the importance of using these tests as ongoing clinical monitoring variables to assess risk profile over time.

Patients who self‐reported strict diets were more likely to have a recent negative IgA TG6 test, supporting that the GFD leads to antibody seroconversion. Self‐reported dietary adherence generally was not associated with clinical outcomes, except for depressive symptoms, where greater adherence was linked to better mood. This study is unable to comment on the cause/effect of such a link. Self‐reported adherence may suffer from biases, and so the possibility of IgA TG6 levels being a more objective and sensitive predictor of outcomes should be considered. Antibody levels may act as useful feedback for patients put on a GFD.

Cognitive testing via the CCAS‐S was administered to some patients, and although this showed a high rate of impairment with less than one‐fifth of participants passing the assessment, performance was not associated with TG6 history. As the longitudinal imaging analyses did show links between IgA TG6 and atrophy, it should be considered that as the cognitive data were a single time‐point, an assessment of change over time with respect to TG6 may be required for a more sensitive analysis. This is highlighted for future study.

Although our findings broadly reflect existing literature, some specifics have not been replicated here. Previous reports have shown brain volume differences in cohorts, with CD to be driven by the presence or persistence of gluten‐related antibodies,^12^, ^30^ but in the present data, exposure to IgA TG6 was only linked to brain atrophy in those patients without CD. However, those previous reports regarded CD patients prospectively recruited via gastroenterology and, therefore, represent a very different population in terms of disease history and referral process. Our baseline scans did not control for pre‐existing structural abnormalities. In any case, the CD subgroup in this study still showed a number of links between TG6 positivity and quality of life outcomes. Furthermore, this is the group where gluten‐driven disease is not a contentious topic; the finding of brain atrophy being associated with TG6 in non‐CD patients arguably holds more value in validating that this can be a serious problem outside of a CD diagnosis.

The study had limitations. The prevalence of TG6 positivity and CD (which was defined by available serological results) may be underestimated if patients were already engaging with a strict GFD at the point of joining the neurology clinic, leading to persistently negative test results. Only including patients with at least 5 years of serological history in major analyses was partially an attempt to control for this, as it places considerable demand on a patient over time to avoid gluten exposures. Restricting patient definitions to a single method based on clinical testing was also seen as advantageous given its consistency and reproducibility. By comparison, examination of patient notes would suffer many of the same issues; for example, with many suspected CD cases already being on a GFD and refusing a gluten challenge during diagnosis. As TG6 testing has only been available since 2019, baseline TG6 serology is lacking for many analyses. This introduces an element of noise, particularly in investigations that define patients by if they have ever had a positive test, and is something that should be addressed by future prospective studies. However, a number of significant results were still found using groups based on positivity, indicating that the injury associated with IgA TG6 remained detectable, despite the potential for false negative cases. Outcomes ascertained from the postal survey may suffer from responder bias. The retrospective nature of this study introduces other limitations. Other clinical covariates of possible interest (eg, apolipoprotein E4 status, cardiovascular risk factors, etc.) were not able to collected systematically or at all. Further research should investigate more granular links with other clinical variables/comorbidities, although the importance of the current investigation in showing for the first time links between IgA TG6 and neurological patients in a real clinical scenario should be stressed. Finally, the study lacked a control group, which limited some certainty around the exact impact of IgA TG6 across different cohorts of people. Ultimately, though, this should not affect confidence in the findings of IgA TG6 being a marker of disease severity in these patients.

In conclusion, this study provided evidence that persistent IgA TG6 antibodies are associated with a variety of poorer brain health outcomes for patients with gluten‐related neurological problems, including accelerated rates of brain atrophy. Together with previous work on newly diagnosed patients with CD, this suggests that their detection is important for stratification of patient care, and their continued monitoring/elimination should be the goal of treatment with the GFD. This applies to all patients with CD and those with gluten‐responsive neurological problems without CD. This study shows that good engagement with the GFD predicts a recent negative IgA TG6 test, supporting the diet as a treatment to ensure good brain health is maintained. Consequently, all relevant patients should be considered for IgA TG6 testing in diagnosis and monitoring, and the GFD should be recommended to those who test positive, even in those without CD.

## Author Contributions

I.C., M.H., D.S., and N.H. contributed to the conception and design of the study. I.C., M.H., K.T., A.B., N.T., and N.H. contributed to the acquisition and analysis of data. I.C. contributed to drafting the text or preparing the figures.

## Potential Conflicts of Interest

M.H. receives royalties from Zedira for TG6 patents, all of which supports research activities.

## Supporting information


**Data S1.** Gluten Sensitivity Questionnaire

**Supplementary Figure S1.** Brain regions where IgA TG6 exposure (ranked) holds a negative correlation with rate of atrophy in patients without CD, corrected for age. Areas highlighted in red/yellow clusters have a faster rate of atrophy with greater IgA TG6 exposure in this group. The template brain used for visualisation is the MNI152 (2 mm) with Z slice co‐ordinates given for reference.

**Supplementary Figure S2.** Scatterplot showing the significant association where increasing IgA TG6 exposure predicts faster rate of yearly atrophy across all regions measured by the “FIRST” analysis (i.e. major basal ganglia and subcortical grey matter areas), in patients without CD and corrected for age.

**Supplementary Table S1.** Findings of analyses replicating primary significant findings in subgroups based on the presence of celiac disease. Findings are bold where significant; some results are replicated in each subgroup or generally otherwise are “approaching” significant (*p* < 0.1).
**Data S2**. Supporting Information

## Data Availability

Study data is not publicly available, as this is not permitted by the ethical approval for the project.
