# Supplementary material for: Persisting Transglutaminase 6 Antibodies in Neurological Gluten‐Related Disorders
Source: Ann Neurol. 2025 Aug 25;99(1):274–82. doi: 10.1002/ana.78020 (PMC12946592; doi:10.1002/ana.78020)
Supplement: Supplementary file 1 — Data S1. Gluten Sensitivity Questionnaire [file ANA-99-274-s002.pdf]

Participant identifier (completed by staff):

Gluten Sensitivity Questionnaire

Completing and returning this questionnaire indicates your consent for this data to be used for research purposes. Personally-identifiable information will not be given to anyone outside of the research team / your medical care team.

Are you happy to be contacted about future research opportunities by staff at the University of Sheffield? Yes ☐ No ☐

Have you completed one of these forms previously? Yes ☐ No ☐

What is your full name? ______________________________

What is your DOB? ____________ **Date form completed?** ___________

What was your biological sex at birth? Male ☐ Female ☐

How long have you had your neurological gluten sensitivity diagnosis?

______ Years ______ Months

Please tick if you have been diagnosed with either of these other gluten-related conditions:

Coeliac Disease ☐

Dermatitis Herpetiformis ☐

Please list other medical conditions you have been diagnosed with; *all* are relevant but please focus particularly on anything *psychological/psychatric* (e.g. mood disorders), *developmental* (e.g. autism spectrum disorder, ADHD), *rheumataological* (e.g. rheumatoid arthritis, fibromyalgia), *gastrointestinal* (e.g. dietary allergies) or *autoimmune* (e.g. psoriasis)

____

____

____

____

____

____

This page aims to assess what symptoms you experience as part of your neurological condition, and in response to gluten. For the following list, please tick all relevant options.

For each symptom you should indicate if you experience it as a "short term" reaction to eating gluten (i.e. that if you ate gluten you would expect that symptom to either appear or get worse than usual), or if you experience it in general (regardless of your diet). Appreciating that many patients will have been on a strict diet for years and might not be sure about "short term" symptoms, please just give these your best guess at what you think would happen from previous experience.

	**As a reaction to gluten**	**Persistently, regardless of diet**
Headaches	☐	☐
Balance problems	☐	☐
Movement problems	☐	☐
Sensory disturbances	☐	☐
Restless legs	☐	☐
Abdominal pain	☐	☐
Diarrhoea	☐	☐
Fatigue	☐	☐
Bloating	☐	☐
Irritability	☐	☐
Constipation	☐	☐
Vomiting	☐	☐
Mouth ulcers	☐	☐
Non-specific skin rash	☐	☐
Brain fog	☐	☐

This page aims to assess your confidence in the gluten-free diet as a treatment for your condition, and also the severity of your symptoms.

For the following questions, please give your answer by striking through the horizontal line at the relevant point. As with the previous page, if you have been on a successful diet for a long time please just give the "short term" symptom questions your best guess from previous experience.

Overall, how effective do you feel the gluten-free diet is at controlling your "short term" symptoms

Not at all |————————————————| *Completely*

OR tick: N/A ☐ (I do not experience short term symptoms after eating gluten)

How effective do you feel the gluten-free diet is at helping your "persistent" symptoms?

Not at all |————————————————| *Completely*

Overall, how severe are your symptoms which happen immediately after eating gluten?

Barely noticeable |————————————————| *As bad as they could be*

OR tick: N/A ☐ (I do not experience short term symptoms after eating gluten)

Overall, how severe are your symptoms which persist regardless of your diet?

Barely noticeable |————————————————| *As bad as they could be*

This section is intended to be a short quiz on your knowledge of the gluten-free diet. Please tick the foods listed below that should not be eaten on a gluten-free diet. Please do not look anything up if you are unsure, and just give it your best attempt.

**Rice** ☐	**Wheat** ☐	
**Barley** ☐	**Mustard** ☐	**Millet** ☐
**Corn** ☐	**Buckwheat** ☐	**Quinoa** ☐
**Soya** ☐	**Rye** ☐	

Regarding your diet *now* (please select only one answer per question)…

	No	Yes – just a taste but rarely	Yes – just a taste and often	Yes – a normal portion
**Do you eat gluten voluntarily?**	☐	☐	☐	☐

	Yes	No
**When you eat out, do you tell the person who is cooking about your disease?**	☐	☐
**Do you check the labels of packaged food?**	☐	☐
**Do you only eat packaged food guaranteed by Coeliac UK?**	☐	☐

Overall, strict is your current gluten-free diet? (strike through line)

I make no effort to restrict gluten |———————————————| *I am as strict as anyone could be*

In the time since your initial diagnosis, have you changed how strictly you approach a gluten-free diet?

I am much less strict than I used to be	I am somewhat less strict than I used to be	No – it has always been the same	I am somewhat stricter now than I used to be	I am much stricter now than I used to be
☐	☐	☐	☐	☐

If there have been periods of time when you have *not* engaged with a strict gluten free diet, please estimate how long <u>in total</u> you have not been gluten-free (since being diagnosed with your initial gluten-related disorder).

______ Years ______ Months OR I have always been on a strict GFD since my initial diagnosis ☐

The last parts of this questionnaire ask more general questions about your health and mental wellbeing.

Hospital Anxiety and Depression Scale (HADS)

Instructions: Doctors are aware that emotions play an important part in most illnesses. If your doctor knows about these feelings he or she will be able to help you more. This questionnaire is designed to help your doctor know how you feel. Read each item and circle the reply which comes closest to how you have been feeling in the past week. Don't take too long over your replies: your immediate reaction to each item will probably be more accurate than a long thought out response.

**I feel tense or 'wound up':**	**A**
Most of the time	3
A lot of the time	2
Time to time, occasionally	1
Not at all	0

**I feel as if I am slowed down:**	**D**
Nearly all of the time	3
Very often	2
Sometimes	1
Not at all	0

**I still enjoy the things I used to enjoy:**	**D**
Definitely as much	0
Not quite so much	1
Only a little	2
Not at all	3

**I get a sort of frightened feeling like 'butterflies in the stomach':**	**A**
Not at all	0
Occasionally	1
Quite often	2
Very often	3

**I get a sort of frightened feeling like something awful is about to happen:**	**A**
Very definitely and quite badly	3
Yes, but not too badly	2
A little, but it doesn't worry me	1
Not at all	0

**I have lost interest in my appearance:**	**D**
Definitely	3
I don't take as much care as I should	2
I may not take quite as much care	1
I take just as much care as ever	0

**I can laugh and see the funny side of things:**	**D**
As much as I always could	0
Not quite so much now	1
Definitely not so much now	2
Not at all	3

**I feel restless as if I have to be on the move:**	**A**
Very much indeed	3
Quite a lot	2
Not very much	1
Not at all	0

**Worrying thoughts go through my mind:**	**A**
A great deal of the time	3
A lot of the time	2
From time to time but not too often	1
Only occasionally	0

**I look forward with enjoyment to things:**	**D**
A much as I ever did	0
Rather less than I used to	1
Definitely less than I used to	3
Hardly at all	2

**I feel cheerful:**	**D**
Not at all	3
Not often	2
Sometimes	1
Most of the time	0

**I get sudden feelings of panic:**	**A**
Very often indeed	3
Quite often	2
Not very often	1
Not at all	0

**I can sit at ease and feel relaxed:**	**A**
Definitely	0
Usually	1
Not often	2
Not at all	3

**I can enjoy a good book or radio or TV programme:**	**D**
Often	0
Sometimes	1
Not often	2
Very seldom	3

SF-12 Health Survey

This survey asks for your views about your health. This information will help keep track of how you feel and how well you are able to do your usual activities. **Answer each question by choosing just one answer**. If you are unsure how to answer a question, please give the best answer you can.

1. **In general, would you say your health is**:

☐1 Excellent ☐2 Very good ☐3 Good ☐4 Fair ☐5 Poor

The following questions are about activities you might do during a typical day. Does your health now limit you in these activities? If so, how much?

	**YES, limited a lot**	**YES, limited a little**	**NO, not limited at all**
2. **Moderate activities** such as moving a table, pushing a vacuum cleaner, bowling, or playing golf.	☐1	☐2	☐3
3. Climbing **several** flights of stairs.	☐1	☐2	☐3

During the past 4 weeks, have you had any of the following problems with your work or other regular daily activities as a result of your physical health?

	**YES**	**NO**
4. **Accomplished less** than you would like.	☐1	☐2
5. Were limited in the **kind** of work or other activities.	☐1	☐2

During the past 4 weeks, have you had any of the following problems with your work or other regular daily activities as a result of any emotional problems (such as feeling depressed or anxious)?

	**YES**	**NO**
6. **Accomplished less** than you would like.	☐1	☐2
7. Did work or activities **less carefully than usual**.	☐1	☐2

8. **During the past 4 weeks, how much did pain interfere with your normal work (including work outside the home and housework)?**

☐1 Not at all ☐2 A little bit ☐3 Moderately ☐4 Quite a bit ☐5 Extremely

These questions are about how you have been feeling during the past 4 weeks.
For each question, please give the one answer that comes closest to the way you have been feeling.

How much of the time during the past 4 weeks…

	All of the time	Most of the time	A good bit of the time	Some of the time	A little of the time	None of the time
9. Have you felt calm & peaceful?	☐1	☐2	☐3	☐4	☐5	☐6
10. Did you have a lot of energy?	☐1	☐2	☐3	☐4	☐5	☐6
11. Have you felt down-hearted and blue?	☐1	☐2	☐3	☐4	☐5	☐6

12. **During the past 4 weeks, how much of the time has your physical health or emotional problems interfered with your social activities (like visiting friends, relatives, etc.)?**

☐1 All of the time ☐2 Most of the time ☐3 Some of the time ☐4 A little of the time ☐5 None of the time
