# Supplementary figures and images for: Persisting Transglutaminase 6 Antibodies in Neurological Gluten‐Related Disorders
Source: Ann Neurol. 2025 Aug 25;99(1):274–82. doi: 10.1002/ana.78020 (PMC12946592; doi:10.1002/ana.78020)

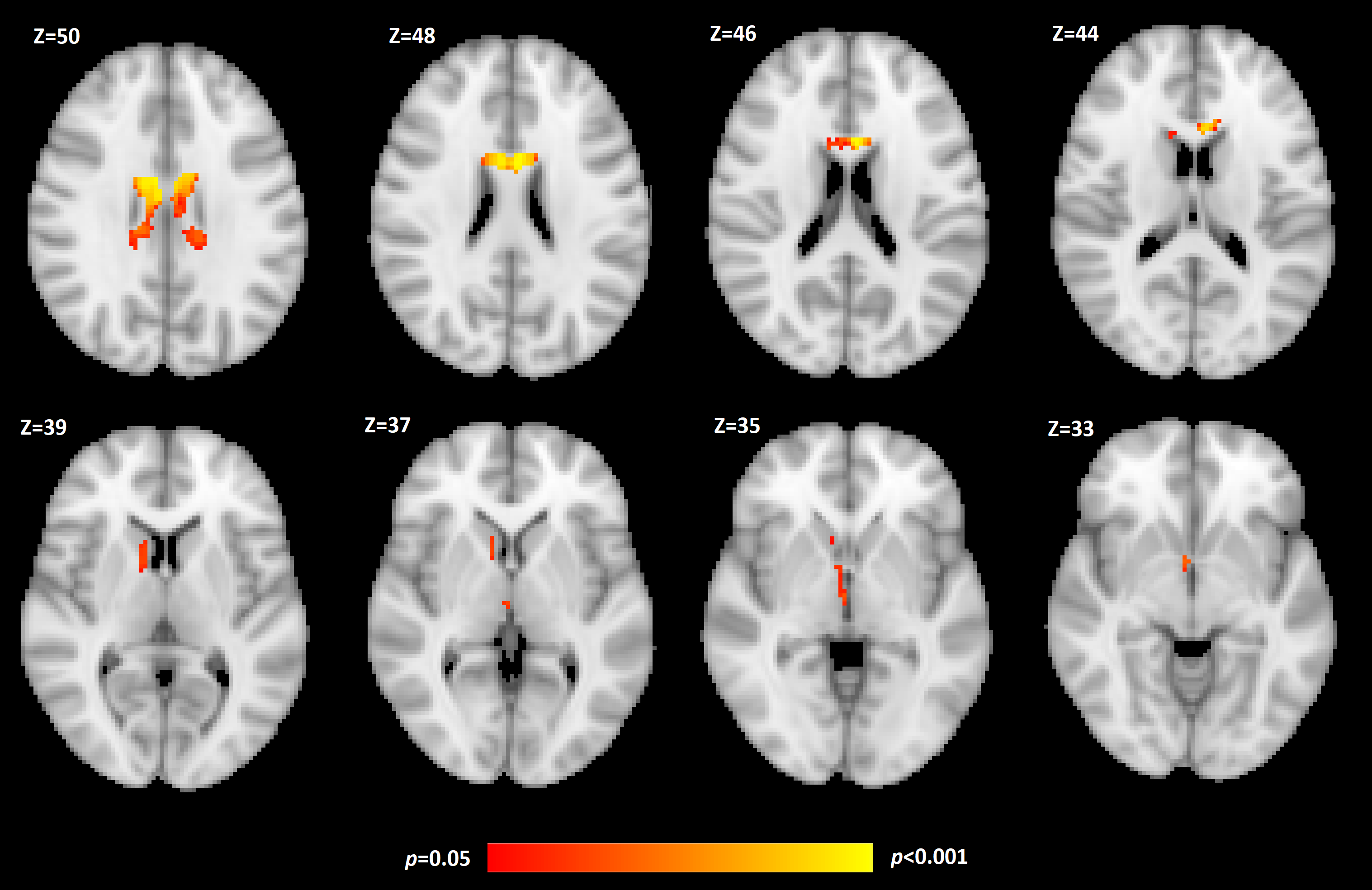

Supplement: Supplementary file 2 — Supplementary Figure S1. Brain regions where IgA TG6 exposure (ranked) holds a negative correlation with rate of atrophy in patients without CD, corrected for age. Areas highlighted in red/yellow clusters have a faster rate of atrophy with greater IgA TG6 exposure in this group. The template brain used for visualisation is the MNI152 (2 mm) with Z slice co‐ordinates given for reference. [file ANA-99-274-s004.tif]

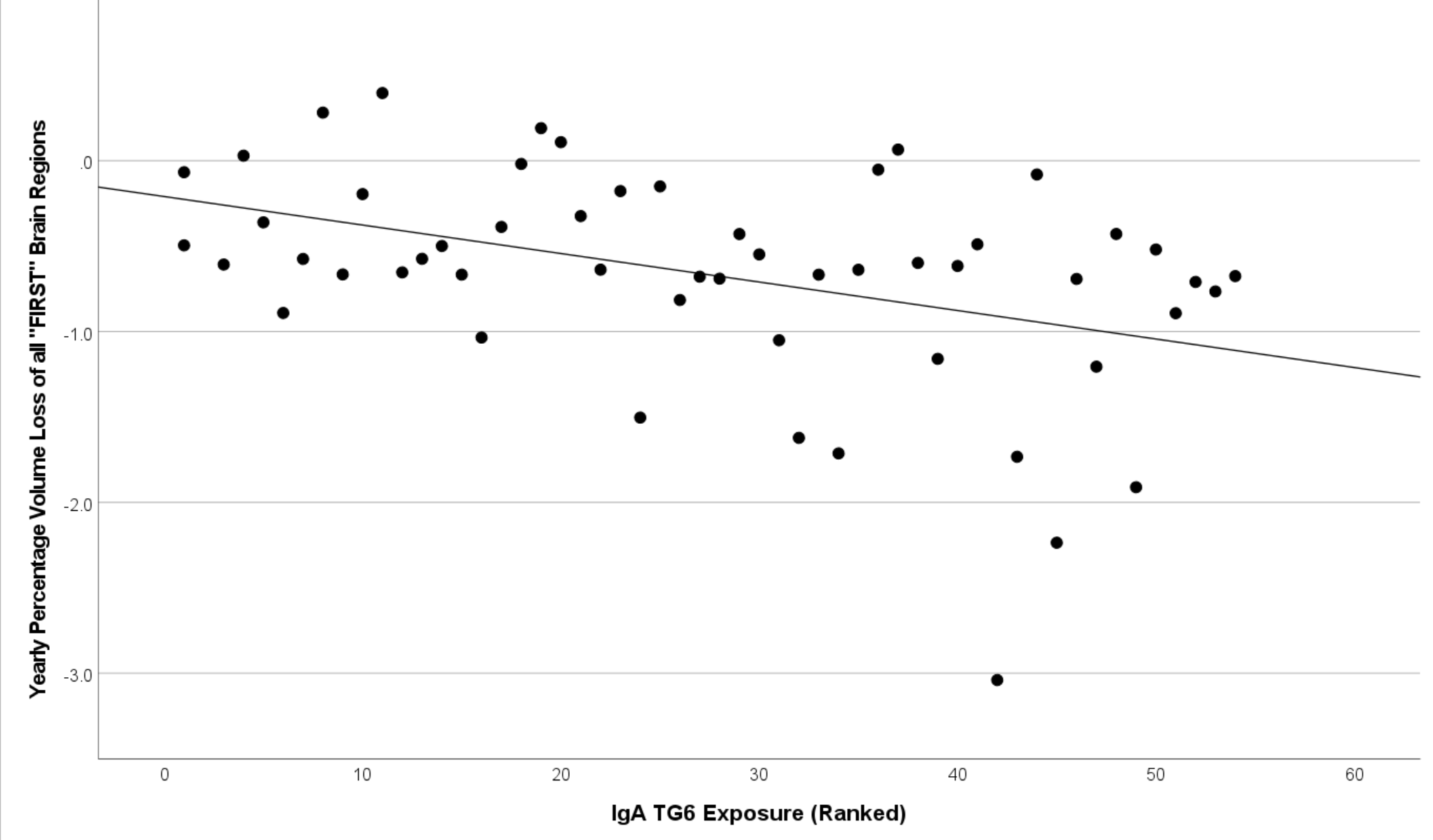

Supplement: Supplementary file 3 — Supplementary Figure S2. Scatterplot showing the significant association where increasing IgA TG6 exposure predicts faster rate of yearly atrophy across all regions measured by the “FIRST” analysis (i.e. major basal ganglia and subcortical grey matter areas), in patients without CD and corrected for age. [file ANA-99-274-s003.tif]
