# Supplementary material for: Persisting Transglutaminase 6 Antibodies in Neurological Gluten‐Related Disorders
Source: Ann Neurol. 2025 Aug 25;99(1):274–82. doi: 10.1002/ana.78020 (PMC12946592; doi:10.1002/ana.78020)
Supplement: Supplementary file 4 — Supplementary Table S1. Findings of analyses replicating primary significant findings in subgroups based on the presence of celiac disease. Findings are bold where significant; some results are replicated in each subgroup or generally otherwise are “approaching” significant (p < 0.1). Data S2. Supporting Information [file ANA-99-274-s001.docx]

**Supplementary Results**

Primary outcome analyses reported in the main paper that are significant across the whole cohort are repeated here using the same data but split into subgroups with and without CD (as defined by there being a positive TG2 or EMA antibody test at any point in serological history). These investigations therefore seek to replicate results and explore if a co-diagnosis of CD drives any findings.

CD and TG6 positivity patterns

The rate of cases (in patients with 5 or more years of serological history) with a positive CD test was 32.7%. X^2^ testing showed the rate of TG6 positivity for either IgA or IgG TG6 was not significantly different when compared between groups based on CD diagnosis.

Questionnaire Outcomes

Across the whole cohort, having at some point a positive IgA TG6 test result significantly predicted poorer chronic symptom severity, HADS depression and SF-12 physical functioning (see Table 1 in the main manuscript). See Supplementary Table 1 below for results replicating these analyses in CD-based subgroups.

| Outcome | **Non-CD**: IgA TG6 positive mean±SD vs. negative mean±SD (*p* value, N) | **CD**: IgA TG6 positive mean±SD vs. negative mean±SD (*p* value, N) |
| --- | --- | --- |
| Chronic symptom severity | 6.3±2.7 vs. 4.7±3.0 (***p*=0.042**, N=51) | 6.2±2.7 vs. 3.5±2.4 (***p*=0.005**, N=44) |
| HADS depression | 7.0±4.5 vs. 4.9±3.0 (*p*=0.076, N=58) | 6.7±3.0 vs. 4.8±3.5 (*p*=0.053, N=46) |
| SF-12 physical functioning | 33.4±13.1 vs. 39.6±11.9 (*p*=0.133, N=50) | 29.2±11.4 vs. 37.9±10.3 (***p*=0.046**, N=39) |

*Supplementary Table 1. Findings of analyses replicating primary significant findings in subgroups based on the presence of celiac disease. Findings are bold where significant; some results are replicated in each subgroup or generally otherwise are “approaching” significant (p<0.1).*

Brain Imaging Outcomes

Across the whole cohort, voxelwise analysis showed IgA TG6 exposure to be significantly correlated with rate of brain atrophy in areas around the corpus callosum and lateral ventricles (in age-corrected models). This analysis was re-performed in subgroups with and without CD. The CD subgroup did not show any significant findings, however the non-CD subgroup found a similar pattern of brain regions to the primary analysis where a faster rate of atrophy was associated with greater IgA TG6 exposure albeit with some additional areas becoming statistically significant with clusters extending into basal ganglia areas (see Supplementary Figure 1).

From this, further analysis was done of the non-CD subgroup. FSL’s “FIRST” pipeline was used to calculate longitudinal volume change of subcortical grey matter areas and the combined rate of atrophy across all these regions also significantly correlated with IgA TG6 exposure where greater exposure predicted a faster rate of atrophy (age-corrected, p=0.020, Supplementary Figure 2).

Subregion-specific analysis showed significant correlations to be present between IgA TG6 exposure and yearly rate of atrophy in the brainstem (*p*=0.045) and accumbens (*p*=0.020). The thalamus and hippocampus similarly showed significant correlations but did not survive age correction.

All significant, age-corrected models (i.e. for all subcortical regions combined, brainstem and accumbens) were re-run with any potential outliers excluded, defined as having a Z score >3 / <-3. In each analysis this resulted in the removal of a single data point, and all results remained significant.

**Supplementary Figure Legends**

*Supplementary Figure 1. Brain regions where IgA TG6 exposure (ranked) holds a negative correlation with rate of atrophy in patients without CD, corrected for age. Areas highlighted in red/yellow clusters have a faster rate of atrophy with greater IgA TG6 exposure in this group. The template brain used for visualisation is the MNI152 (2mm) with Z slice co-ordinates given for reference.*

*Supplementary Figure 2. Scatterplot showing the significant association where increasing IgA TG6 exposure predicts faster rate of yearly atrophy across all regions measured by the “FIRST” analysis (i.e. major basal ganglia and subcortical grey matter areas), in patients without CD and corrected for age.*
